# Polymorphs and Prodrugs and Salts (Oh My!): An Empirical Analysis of “Secondary” Pharmaceutical Patents

**DOI:** 10.1371/journal.pone.0049470

**Published:** 2012-12-05

**Authors:** Amy Kapczynski, Chan Park, Bhaven Sampat

**Affiliations:** 1 Yale Law School, Yale University, New Haven, Connecticut, United States of America; 2 Medicines Patent Pool, Geneva, Switzerland; 3 Department of Health Policy and Management, Mailman School of Public Health, Columbia University, New York, New York, United States of America; University of British Columbia, Canada

## Abstract

**Background:**

While there has been much discussion by policymakers and stakeholders about the effects of “secondary patents” on the pharmaceutical industry, there is no empirical evidence on their prevalence or determinants. Characterizing the landscape of secondary patents is important in light of recent court decisions in the U.S. that may make them more difficult to obtain, and for developing countries considering restrictions on secondary patents.

**Methodology/Principal Findings:**

We read the claims of the 1304 Orange Book listed patents on all new molecular entities approved in the U.S. between 1988 and 2005, and coded the patents as including chemical compound claims (claims covering the active molecule itself) and/or one of several types of secondary claims. We distinguish between patents with any secondary claims, and those with only secondary claims and no chemical compound claims (“independent” secondary patents).

We find that secondary claims are common in the pharmaceutical industry. We also show that independent secondary patents tend to be filed and issued later than chemical compound patents, and are also more likely to be filed after the drug is approved. When present, independent formulation patents add an average of 6.5 years of patent life (95% C.I.: 5.9 to 7.3 years), independent method of use patents add 7.4 years (95% C.I.: 6.4 to 8.4 years), and independent patents on polymorphs, isomers, prodrug, ester, and/or salt claims add 6.3 years (95% C.I.: 5.3 to 7.3 years). We also provide evidence that late-filed independent secondary patents are more common for higher sales drugs.

**Conclusions/Significance:**

Policies and court decisions affecting secondary patenting are likely to have a significant impact on the pharmaceutical industry. Secondary patents provide substantial additional patent life in the pharmaceutical industry, at least nominally. Evidence that they are also more common for best-selling drugs is consistent with accounts of active “life cycle management” or “evergreening” of patent portfolios in the industry.

## Introduction

Patents play a distinctively important role in the global pharmaceutical industry today. Studies suggest that pharmaceutical firms consider patents critical to their efforts to recoup R&D investments, much more so than firms in other industries [1]–[3]. This is believed to reflect the difference between the high cost of discovering and testing new drugs and the low cost of reverse engineering generic copies of existing drugs [4]. The flip side of this is that once drug patents expire, generic competition can reduce prices [5] and promote wider access to medicines.

Though the pharmaceutical industry is often cited as the epitome of a “discrete product” industry – one with low patent-product ratios [2] – the number of patents per new drug has grown dramatically over the past three decades [6]. Part of this growth presumably reflects the many types of claims now common in the pharmaceutical sector. Medicine products may be associated, for example, not only with patents covering the base compound. They may also be covered by patents covering modified forms of that base compound, medical uses of a known chemical compound, combinations of known chemical compounds, particular formulations (tablets, topical forms), dosage regimens, and processes, among others [6]. This paper examines the rise of these “secondary” patents, and assesses their impact on patent life. These patents are generally termed secondary because they are assumed to come later in the sequence of innovation, and to offer less robust protection than a chemical compound claim. We use the term not because we believe these patents to be necessarily of lesser importance or strength, but because the term is conventional in the literature, and among practitioners [6]–[8].

Secondary patents are interesting for several reasons. First, much of the literature on pharmaceutical patents focuses on primary patents, making secondary patents under-studied. For example, in several influential studies of effective patent life in the pharmaceutical sector, Grabowski and Vernon estimate that delays related to the regulatory review process lead to effective patent terms of approximately ten or eleven years [9]. Figures such as these are widely reproduced in the literature, and used to justify patent term extensions and other supplemental forms of market exclusivity [8], [10]. Yet they have a notable shortcoming: they compute patent life based on the primary patents available, and generally ignore secondary patents [9]. If secondary patents are frequently obtained later in the invention cycle than chemical compound patents, this will underestimate patent life, perhaps substantially. However to our knowledge there are only a few large-sample empirical studies of secondary patents [11].

A better understanding of secondary patenting is important, because these patents are perceived by pharmaceutical practitioners as critical to practices of “life cycle management,” and thus to business strategy. As one recent article put it: “A key element of any life cycle management strategy … is to extend patent protection beyond the basic patent term for as long as possible, by filing secondary patents which are effective to keep generics off the market” [7]. Secondary patents also may be becoming more important to industry over time, particularly if declining R&D productivity [12] puts more pressure on companies to extract profit from existing drugs. In a recent study by the Organization for Economic Cooperation and Development, more than 85% of the pharmaceutical companies surveyed reported an increase in patenting activity over ten years before, and many attributed this in part to new efforts to patent discoveries that they would not have sought to patent ten years before, even if they were patentable then [13]. A recent European Commission report offers a compatible account from a pharmaceutical executive, who characterized the situation prior to the end of the 1980s as one where products were “mainly [chemical entities] which where protected by the one patent,” and the period of the late 1980s to early 1990s as one characterized by “[e]xpansion of the portfolio to cover lifecycle initiatives, to extend protection time for product and the brea[d]th of the protection trying to keep competition further away” [6].

The flip side of this is the widespread allegation that secondary patents are part of firms' “evergreening” strategies to extend monopoly protection on existing products [10]. The term evergreening is used to refer to a range of practices. Some are independent of patent strategy [14]–[16], but others depend importantly on secondary patents. For example, such patents may be listed on the FDA's Orange Book and thus can provide opportunities for automatic injunctions against generic competitors [11].

Secondary patents are also interesting because some have argued that certain secondary claims lack true inventiveness and should not be granted [17]. Patents on new medical uses have at various points been controversial in Europe, because of the European Patent Convention's exclusion of patents on methods of medical treatment [10]. Many developing countries outright forbid patents on new uses of known substances [10]. Patents on enantiomers of known racemic chemical compounds, as well as “pure forms” of known substances, have been viewed as obvious or non-novel [10], [17]. Concern about non-innovative secondary patents in pharmaceuticals reflects a broader one, across industries, that resource constrained patent offices may be issuing a large number of low quality patents, i.e. patents that would not have been granted if subjected to proper scrutiny [18].

Not surprisingly, policymakers are also interested in the implications of secondary patents. For example, a recent European Commission inquiry considered the role of secondary patents in the pharmaceutical sector, and concluded that some originator companies appear to use them specifically to inhibit generic competition, despite the fact that they are perceived as generally weak patents [6]. Similar questions have been raised by the U.S. FTC [19]. Recent court decisions may make secondary patents more difficult to obtain in the U.S., with unknown implications for the industry [8], [20]. Secondary patents are also at the center of current controversy in India, where the new patent law excludes patents on new uses, combinations, and new forms of known substances that do not increase efficacy [21]. India's example has recently been followed by several other developing countries [21].

How big of a difference would policies restricting secondary patents make to the patent landscape in pharmaceuticals? What precisely might be at stake if patent standards are strengthened in a way that casts doubt over certain classes of secondary patents? The answer to these questions, and the other policy questions discussed above, requires information on the prominence and impact of secondary patenting in pharmaceuticals, currently lacking. This paper aims to begin to fill this void. We provide novel data that aims to address the following questions: What is the prevalence of patents with chemical compound claims, patents with secondary claims, and of “independent” secondary patents that have no chemical compound claims? When are chemical compound and independent secondary patents filed, relative to drug approval? What are the effects of these patents on the patent life? And does the prevalence of independent secondary patenting vary with sales?

## Data and Methods

### Drug data

We began by collecting data on the 528 new molecular entities (NMEs) approved by the U.S. Food and Drug Administration between 1988 and 2005, from the *Drugs@FDA* database. According to FDA definitions, NMEs are drugs where the active ingredient was not previously approved by the agency [22].

### Sales

To examine how propensity to obtain secondary patents varies with drug sales, we also generated national estimates of sales for the drugs in our sample based on information from the Prescribed Medicines File of the Medical Expenditure Panel Survey [23]. We obtained these data annually for the 1996–2010 period.

We are interested in the effects of sales on propensity to obtain different types of patents. One difficulty is that patents may also affect drug sales, for example, by preventing generic entry. Accordingly, we use sales estimates from a point in the drug's lifecycle when generic entry is not possible: in the fifth year after a drug is approved. Before this time, generic competition is typically not possible even absent patents for most NMEs, due to “data exclusivity” restrictions on generic entry. (Data exclusivity is the term for exclusive marketing rights that stem not from the patent system, but from the drug regulatory system. Some countries, such as the U.S., award periods of data exclusivity upon the submission of certain clinical trial data.) Previous analysis [24] of first time generic entry on NMEs over the 2001–2010 period shows no instances of generic entry before the fifth year after NME approval.

Since our sales data are for 1996–2010, collection of sales in year five means that in analyses of sales, we limit the sample to the 342 NMEs (that have at least one patent) that were approved between 1991 and 2005. We adjusted sales to 2010 constant dollars using the commodities PPI (Producer Price Index) deflator.

In the analyses of sales, we group drugs by sales quartiles. This allows us to examine the relationship between sales and patenting with a flexible functional form, particularly important in this industry since high-selling “blockbuster” drugs are known to have different patent dynamics than others [24]. It also reflects the practical reality that MEPS sales figures are estimates based on a sample, and we cannot determine whether drugs with no reported sales in MEPS actually have zero sales, or instead have low sales that MEPS lacks the statistical power to detect. The bottom sales quartile thus captures “low or no sales” drugs.

### Orange book patent data

We combine the drug approval data with patent data from another FDA database, the Orange Book, a compendium of patents pertinent to approved drugs based on information provided to the agency by the originator firms [25]. We compiled data on patents on drugs from the machine-readable versions of the FDA's Orange Book (“the Electronic Orange Book”) released between 2000 and 2009. Since recent versions of the Orange Book list only unexpired patents, we also obtained a file with information on all expired patents (from pre-2000 versions) from the FDA, via a Freedom of Information Act request. (We verified the FOIA data on older expired patents against printed copies of previous Orange Books published from 1988–2000, and found the sources to be substantially in agreement, with the exception of what appear to be transcription errors in the printed versions.) For each drug, we thus collected information on all expired and unexpired patents that were listed in the Orange Book.

Overall, the 528 NMEs map to 1261 distinct patents, and 1304 total patents. (On occasion, a patent can be associated with multiple NMEs. For example, the process patent 4,396,597 is listed on the Orange Book for two drugs Omnipaque (iohexol) 180, approved in 1985, and Visipaque (iodixanol) 270, approved in 1996.) And some NMEs (96) have no patents on the Orange Book: drugs without patents tend to rely on other forms of FDA exclusivity for market protection, e.g. Orphan Drug exclusivity.) Since our focus is on patenting, we exclude these drugs from our analysis.

### Expiration dates

From the Orange Book, we determined the expiration dates for each of the patents. Orange Book listings reflect the statutory term, as well as patent extensions or patent term adjustments. Since expiration dates for patents sometimes change over time (e.g. via grant of special exclusivity periods such as those offered in compensation for pediatric trials) we take the maximum expiration date for each drug-patent observation.

### Application and issue dates

We obtained information about the application date and issue dates for each of the patents from the United States Patent and Trademark Office's *Cassis* database of issued patents [26].

### Patent coding

We read through each claim in these patents, and determined whether the patent had one or more of the following types of claims:

- *Chemical compound* claims: those claiming an active ingredient that had not previously been disclosed in the art.
- *Formulation* claims: those directly onclaiming specific pharmaceutical preparations to administer a product (e.g. tablets, dosage forms, sustained release forms)
- *Method of treatment/use* claims: methods of treating specific diseases or conditions with particular compounds
- *Polymorph, Isomer, Prodrug, Ester, Salts (“PIPES”)* claims: minor modifications of the structure or chemical makeup of a molecule

To do so, two of the authors [CP and AK] initially each independently coded several hundred of the same patents to clarify the categories and then divided the sample (with each patent coded by one person). As a check on accuracy, patents with complex claims (typically, those claiming either a compound or a new form of a known compound) were re-coded by research assistants with doctoral degrees in chemistry and experience in pharmaceutical patenting.

The Appendix S1 discusses the categories and coding rules in detail.

Chemical compound claims represent primary claims, and the other three categories secondary claims. An individual patent can (and often does) have claims from more than one of these categories. For example, the first filed patent on a drug sometimes has chemical compound claims as well as one or more types of secondary claims. To distinguish between such patents and those with purely secondary claims, we also determined which patents were “independent” secondary patents, those with secondary claims only. We make this distinction since patents with independent secondary claims are those that are most important for discussions of evergreening, since secondary claims in patents that also have chemical compound patents do not generate additional patent life.

Under this definition an “independent formulation patent” can also have other types of secondary claims, e.g. PIPES claims. Note also that we use the term “secondary” to differentiate these claims/patents from primary (chemical compound) claims. Their characterization as “secondary” is not meant to imply anything about their temporal relationship to chemical compound patents, though we will show that independent secondary patents are generally obtained later in the product life cycle.

For drugs with an independent secondary patent we calculated incremental patent life generated by each such patent. In cases where a drug has a chemical compound patent and an independent secondary patent of a particular category, the incremental life associated with that category is defined as the difference between expiration date of the last expiring patent in that secondary category and the last expiring chemical compound patent. Where there is no chemical compound patent, incremental life is defined as the difference between the expiration of the last expiring patent in that secondary category and the expiration of the regulatory exclusivity period for the drug (in the U.S., generally five years after the drug is approved) after which generic entry can commence. The logic behind these measures is to examine the incremental life generated by independent secondary patents, as compared to that sustained by other forms of protection (chemical compound patents or regulatory exclusivity).

As we note in the Table S1, other types of claims–process claims, product-by-process claims, medical device claims, particle sizes, combinations, and pure forms–were coded but excluded from analyses, since they are individually small in number. Since some of these categories are reasonably considered secondary claims, our analyses below provide conservative estimates of the prevalence of secondary patenting. In the discussion we offer additional reasons that our method is likely to understate, rather than overstate, the importance of secondary patents.

### Analytical methods

Using these sources and measures, we determined (1) the share of drugs with chemical compound patents and different types of secondary claims; (2) the share of drugs with each type of independent secondary patent; (3) the share of drugs with chemical compound patents and different types of secondary claims, by approval year; (4) the share of drugs with each type of independent secondary patent, by approval year; (5) the timing of filing/issue of chemical compound patents and each type of independent secondary patent; (6) average incremental patent life generated by each type of independent secondary patent; and (7) the share of drugs that have chemical compound patents and independent secondary patents, by sales quartile. In addition to providing descriptive data, we estimate logit regressions relating independent secondary patenting to sales, controlling for application year effects.

## Descriptive Results

### The prevalence of secondary patenting

The first column of results in Table 1 shows the share of drugs with patents that have claims in a given category. Less than two-thirds of the drugs have chemical compound claims in one or more of their patents. This reflects that the active substance in a drug can be “new” to the FDA (never before approved for use in humans) yet known in the chemical arts. Formulation and method of treatment/use claims are quite prevalent, and the share of drugs with such claims is higher than the share with chemical compound claims. PIPES claims are less common, but still present in about half of all drugs. The second column shows similar trends in the average number of patents per drug, by category.

**Table 1:** Chemical compound and secondary patents for drugs in sample.

Category	Number (share) of the 432 drugs with patents that have claims in this category	Average of the number of patents per drug with claims in this category, calculated across the 432 drugs ± standard deviation	Number (share) of the 432 drugs with at least one independent secondary patent in this category	Average of the number independent secondary patents in this category per drug, calculated across the 432 drugs ± standard deviation
Chemical compound	278 (64%)	.85±.84	N/A	N/A
Formulation	348 (81%)	1.6±1.4	242 (56%)	.99±1.24
Polymorph, Isomer, Prodrug, Ester, Salts (“PIPES”)	219 (51%)	.74±.91	104 (24%)	.33±.68
Method of use	357 (83%)	1.8±1.7	272 (63%)	1.3±1.6

Legend: Based on the 432 new molecular entities (with at least one patent) approved by the U.S. Food and Drug Administration between 1985 and 2005. Categories are based on authors' coding of the claims in the 1304 patents (1261 distinct patents) associated with these drugs. “Independent” secondary patents are those that do not have chemical compound claims.

The third results column focuses on independent secondary patents, i.e. patents with secondary claims and no chemical compound claims. The majority of the drugs have independent secondary formulation or method of treatment/use patents, while nearly a quarter have standalone PIPES patents. (Recall there is double-counting across the last three categories, so a patent with both formulation and PIPES claims would count as both.) The final column shows similar trends in the average number of independent secondary patents per drug, by category.

**Figure 1:**
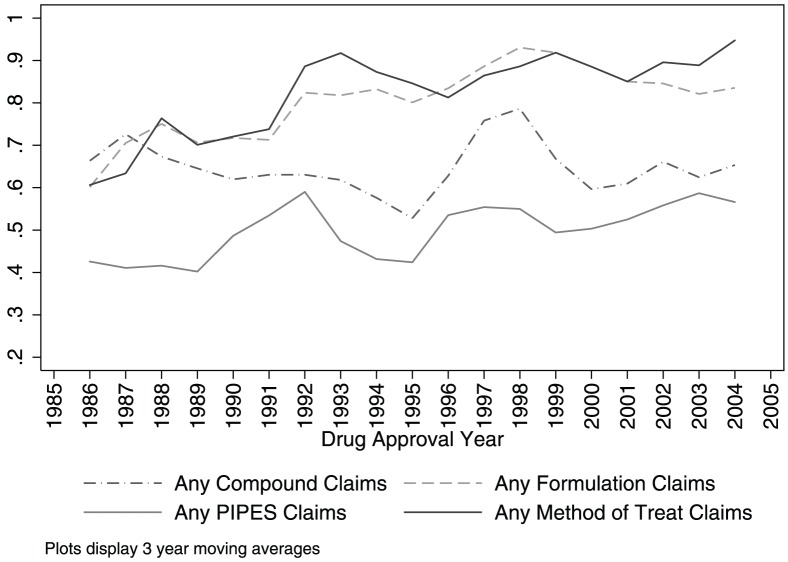
Share of drugs with chemical compound and secondary patent claims by approval year (three year moving averages). Based on the 432 new molecular entities (with at least one patent) approved by the U.S. Food and Drug Administration between 1985 and 2005. Categories are based on authors' coding of the claims from the 1304 patents (1261 distinct patents) associated with these drugs. “PIPES” refers to Polymorph, Isomer, Prodrug, Ester, and Salt claims. The horizontal axis is drug approval year. The vertical axis measures the moving average of the share of drugs in an approval year with at least one patent in a category.

Figure 1 shows that the share of drugs with chemical compound claims in one or more of their patents, by drug approval year, is fairly constant over time. The share of drugs with chemical compound claims is about 65 percent in both the first three years of our sample (1985–1987) and the last three years (2003–2005). Across these same cohorts, the share with formulation claims increased from 60 percent to 84 percent, the share with PIPES claims increased from 43 percent to 57 percent, and the share with method of treatment claims increased from 61 percent to 95 percent.

**Figure 2:**
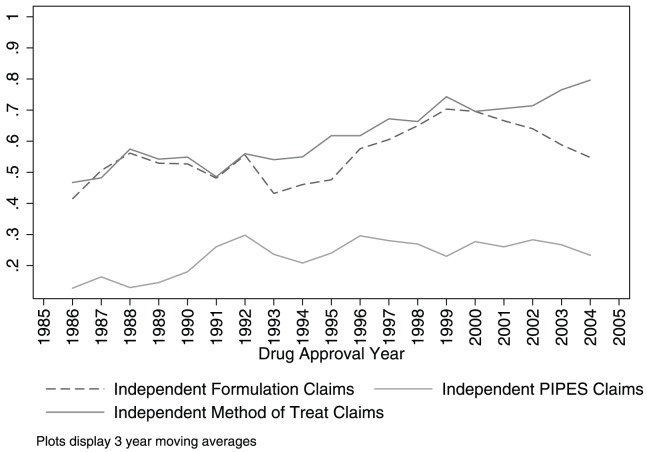
Share of drugs, by approval year, with independent secondary patents. Based on the 432 new molecular entities (with at least one patent) approved by the U.S. Food and Drug Administration between 1985 and 2005. Categories are based on authors' coding of the claims from the 1304 patents (1261 distinct patents) associated with these drugs. “PIPES” refers to Polymorph, Isomer, Prodrug, Ester, and Salt claims. “Independent” secondary patents are those with no chemical compound claims. The horizontal axis is drug approval year. The vertical axis measures the moving average of the share of drugs in an approval year with at least one patent in a category.

Figure 2 focuses on independent secondary patents. It illustrates that the share of drugs with independent formulation patents has increased over time, as has the share with independent PIPES patents and independent method of treatment/use patents. Comparing the same cohorts as above, the share of drugs with independent formulation patents increased from 41 percent to 55 percent, the share with independent PIPES patents from 13 to 23 percent, and the share with independent method of treatment/use claims from 47 to 80 percent.

### Timing of independent secondary patents

Above we have used the term independent secondary patents to refer to those with formulation, PIPES, and/or method of treatment claims but not chemical compound claims. We also looked at when the patents were filed (and issued) relative to marketing approval, to see if they are, in fact “secondary” in the sense of later-in-time. Table 2 shows the share of chemical compound patents and independent secondary patents in each category that are filed or issued after the drug is approved. Consistent with our expectations, the vast majority of chemical compound patents are filed and issued before FDA approval. By contrast, a higher share of independent secondary patents is filed after approval, and about 46 percent (overall, across all types of secondary claims) issue after approval.

**Table 2:** Timing of patent filing and issue relative to drug approval, by category.

	Number (share) filed post approval	Number (share) issued post approval
Chemical compound patents (n = 364)	6 (2%)	40 (11%)
Independent formulation patents (n = 430)	91 (21%)	201 (47%)
Independent PIPES patents (n = 143)	34 (24%)	67 (47%)
Independent method of use patents (n = 579)	130 (23%)	261 (45%)

Legend: Based on the patents associated with the 432 new molecular entities (with at least one patent) approved by the U.S. Food and Drug Administration between 1985 and 2005. Categories are based on authors' coding of the claims in these patents. “PIPES” refers to Polymorph, Isomer, Prodrug, Ester, and Salt claims. “Independent” secondary patents in a category are those with no chemical compound claims.

### Effects of secondary patents on patent term

That independent secondary patents tend to be filed and issued later raises the possibility that they may be important in extending the total exclusivity period for drugs, since patent term in the U.S. runs from the longer of 20 years from application or 17 years from issue for pre-1995 patents, and 20 years from application for post-1995 patents.

Table 3 shows that each of these types of independent secondary patents is associated with additional nominal patent term. The first column of results shows that these patents, where they are present, generate between four and five years of additional patent life beyond chemical compound patents, on average. For the drugs without chemical compound patents but with independent secondary patents (about one-third of drugs in the sample), the incremental life is larger, not surprisingly. Across all drugs, those with and without chemical compound patents, the average increment ranges from 6.3 years (for PIPES patents) to 7.4 years (for use patents).

**Table 3:** Average incremental patent life from independent secondary patents, by type.

	Drugs with chemical compound patents	Drugs without chemical compound patents	All drugs
Average incremental patent life from independent formulation patents	4.7 years	9.3 years	6.5 years
N	140	102	242
95% C.I.	3.8–5.5 years	8.3–10.4 years	5.9–7.3 years
Average incremental patent life from independent PIPES patents	4.7 years	8.7 years	6.3 years
N	62	42	104
95% C.I.	3.4–5.9 years	7.2–10.3 years	5.3–7.3 years
Average incremental patent life from independent method of use patents	4.9 years	10.5 years	7.4 years
N	151	121	272
95% C.I.	4.1–5.7 years	8.6–12.4 years	6.4–8.4 years

Legend: Based on the 432 new molecular entities (with at least one patent) approved by the U.S. Food and Drug Administration between 1985 and 2005. (Overall 276 have a chemical compound patent, and 156 do not.) Categories are based on authors' coding of the claims in the 1304 patents (1261 distinct patents) associated with these drugs. “PIPES” refers to Polymorph, Isomer, Prodrug, Ester, and Salt claims. “Independent” secondary patents in a category are those with no chemical compound claims. For each row, incremental patent life is calculated only for drugs with at least one patent in the category, i.e. the figures represent average incremental patent life from each type of secondary patent conditional on having at least one patent of that type.

### Sales

Finally, we examined how the propensity to obtain chemical compound patents and independent secondary patents varies by the branded drug's sales. Since we are interested in how sales affect the propensity to obtain these patents, we focus on independent secondary patents filed after the branded drug was approved, after which market expectations are more certain.

**Figure 3:**
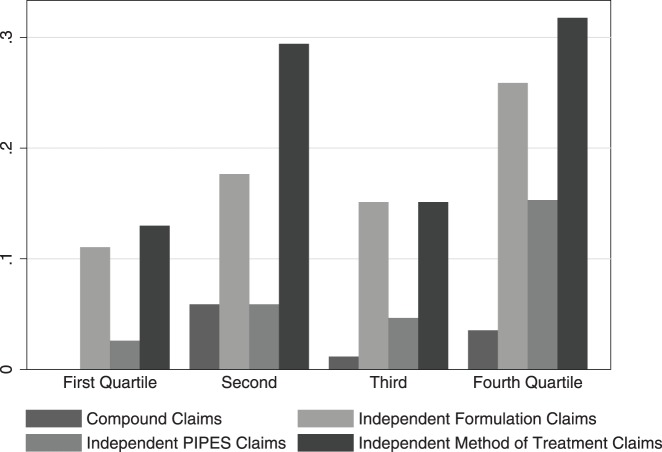
Share of drugs, by sales quartile, with chemical compound and independent secondary patents that are filed after drug approval. Based on the 342 new molecular entities (with at least one patent) approved by the U.S. Food and Drug Administration between 1991 and 2005. Categories are based on authors' coding. “PIPES” refers to Polymorph, Isomer, Prodrug, Ester, and Salt claims. Independent patents are those with no chemical compound claims. Sales categories are based on national estimates of sales (using information from the Medical Expenditure Panel Survey) in the fifth year after brand drug approval. The horizontal axis is quartile of sales. The vertical axis is the share of drugs in a cohort with at least one patent in a category.

Figure 3 shows the share of drugs that have one or more (post-approval) patents in a given category. Overall, and consistent with the patent level analyses in Table 2, the figure shows that few drugs have patents with chemical compound claims that were filed after drug approval, and there is only a slight increase over the sales distribution, from none in the first quartile to 3.5 percent in the top quartile. There is a sharper increase in firms' propensity to obtain independent secondary patents for higher sales drugs. The share of drugs with independent (post-approval) formulation patents increases from 11 percent to 26 percent between the bottom and top sales quartiles. Similarly, the share of drugs with independent PIPES patents (3 percent versus 15 percent) and independent method of treatment/use claims (13 percent versus 32 percent) increases between the bottom and top sales categories.

### Regression analyses

In addition to the descriptive analyses, we examine the effects of sales on post-approval independent secondary patenting through logit regression models. These models relate whether a drug has any post-approval claims of a given type to sales categories and approval year. Table 4 shows the results. In each of the models drugs in the top sales category are significantly more likely to have such patents than the left out category (the bottom sales quartile), and the point estimates are largest for the top quartile, suggesting that post-approval secondary patenting is most common for the highest sales drugs. While logit coefficients are difficult to interpret directly, marginal effects calculations (from Model 4) show that drugs in the top sales category have an 18 percentage point higher likelihood of having any post-approval independent secondary patents than the left out category.

**Table 4:** Logit models relating whether a drug has post-approval independent secondary patent claims to sales and approval year.

	(1)	(2)	(3)	(4)
	Independent Formulation Claims?	Independent PIPES Claims?	Independent Method of Treatment Claims?	Any Independent Secondary Claims?
Second Sales Quartile	0.553	0.861	1.038	0.509
	(0.688)	(1.151)	(0.586)	(0.571)
Third Sales Quartile	0.345	0.584	0.159	0.233
	(0.397)	(0.721)	(0.386)	(0.322)
Top Sales Quartile	1.014**	1.883**	1.117***	0.906**
	(0.358)	(0.591)	(0.336)	(0.301)
Approval Year	−0.0453	−0.0665	−0.0499	−0.0410
	(0.0382)	(0.0577)	(0.0360)	(0.0319)
Constant	88.38	129.2	97.74	80.48
	(76.37)	(115.2)	(71.96)	(63.76)
N	342	342	342	342

Notes:

* indicates p<.05;

** p<.01;

*** p<.001; Standard errors in parentheses. Based on the 342 new molecular entities (with at least one patent) approved by the U.S. Food and Drug Administration between 1991 and 2005. Categories are based on authors' coding. “PIPES” refers to Polymorph, Isomer, Prodrug, Ester, and Salt claims. Independent patents are those with no chemical compound claims. Sales categories are based on national estimates of sales (using information from the Medical Expenditure Panel Survey 1996–2010) in the fifth year after brand drug approval. The left-out category is the bottom sales quartile.

The coefficient on the approval year variable is negative and not statistically significant, suggesting no significant time trend. This may reflect that the increase in independent post-approval secondary patenting occurs before 1991 (the first year when we have sales data); it could also reflect that these patents are particularly susceptible to censoring since they are filed relatively late in a drug's lifecycle. Supplementary analyses, available on request, show that these results, and the estimated effects of sales, are robust to including indicators for each approval year instead of a linear time trend.

## Discussion

Although secondary patents are often criticized, they are rarely studied. We here offer an analysis of the role and effect of secondary patents in the pharmaceutical industry, in an effort to help inform the important policy debates that surround these patents. We do not attempt here to mediate between those who favor and oppose secondary patents of different types, but instead offer an empirical picture of what is at stake in these debates.

We show, first, that patents with secondary claims are extremely common – indeed, more common than chemical compound patents, for the new molecular entities in our sample. While it is sometimes assumed that a new active ingredient is associated with a chemical compound patent, for example, we show that if an NME is associated with a patent (which the vast majority are), it is more frequently associated with a formulation patent (81% of drugs) or a method of treatment/use patent (83%) than with a chemical compound patent (64%). Patent claims covering new forms of known substances (PIPES) are also very common, present in half of all drugs (51%).

Moreover, independent secondary patents tend to come later than primary patents. We measure both forms of patents against the baseline of drug approval, and find that effectively all chemical compound patents are filed before drug approval, and early enough that only 11% issue after the approval date. By contrast, secondary patents tend to be filed later, with nearly one in five secondary patents filed after the drug is approved by the FDA, and close to half issuing after the approval date.

Independent secondary patents on average add substantial time to the nominal patent terms enjoyed by drugs. For drugs that have chemical compound patents, secondary patents add on average between 4 and 5 years of additional nominal patent term. Drugs that do not have chemical compound patents rely much more substantially on secondary patents for exclusivity: here, when there are secondary patents, they generate an average of 9 and 11 years of patent term beyond the standard data exclusivity period.

Moreover, our analysis of patents filed after drug approval reveals that independent secondary patents are not randomly distributed. Firms' propensity to obtain independent secondary patents after drug approval increases over the sales distribution, suggesting they reflect deliberate attempts by branded firms to lengthen their monopoly for more lucrative drugs.

Our results are particularly notable because our sample and data are structured to minimize the importance of secondary patents. Most importantly, our sample only includes NMEs. While we have not analyzed this here, we believe non-NMEs (e.g. line extensions) are less likely to have chemical compound patents, and thus are more reliant on secondary patents. Second, as noted above we observe a rise in secondary patenting over time despite censoring. Third, our data excludes certain kinds of secondary patents: in particular, process patents are not listed on the Orange Book. Industry representatives suggest that process patents may play an important role in life-cycle management strategies [7]. A recent analysis of secondary patents on two antiretroviral drugs reports a large number of unlisted patents, including but not limited to process patents [27].

One factor that our analysis does not incorporate is litigation. Secondary patents may be more vulnerable to attack than chemical compound patents, and if they are frequently invalidated or designed around, they will in practice have less effect on market exclusivity than their effects on nominal patent life suggest [11], [24]. There is reason to suspect that this is the case. Although industry groups reject the suggestion that secondary patents are weaker than chemical compound patents, in practice companies that seek such patents often appear to hold this view [6]. Previous empirical work shows that drugs with non-active ingredient patents, particularly those that generate incremental patent life, are much more likely to attract patent challenges in the U.S. [11], [24]. A European Commission study of the sector recently concluded that generic litigation “mainly concerns secondary patents,” and that generic companies have high success rates in cases involving secondary patents [6].

Even if secondary patents are perceived (and perceived correctly) as more vulnerable than chemical compound patents, this does not mean that they are without meaningful effects. A patent that is ultimately invalidated could still yield substantial benefits for an originator company. Patent litigation in the pharmaceutical industry is notoriously risky and resource intensive, and becomes more so where more patents and claims are involved. This reduces the potential pool of competitors to those with the resources to wage multi-year patent battles. Such litigation may take several years to resolve (the European Commission [6] estimates almost three years for an average case) and in the U.S. a secondary patent may provide the basis for an automatic 30-month stay on generic approval under the Hatch-Waxman Act. This again comports with anecdotal reports from the industry, such as this one expressed by a pharmaceutical executive from an originator company: “Secondary patents will not stop generic competition indefinitely but may delay generics for a number of years, at best protecting the originator's revenue for a period of time” [6]. It is possible that even a weak secondary patent that is invalidated after litigation could produce years of valuable exclusivity, though this is ultimately an empirical question.

Furthermore, litigation as a means to invalidate weak secondary patents is a far less plausible policy outcome in countries without robust incentives for generics to undertake the expense of challenging these patents. Insofar as the policy response to the rise of secondary patents relies on litigation and rigorous patent examinations as a means to ensure that only truly inventive secondary patents issue, resource-limited settings are likely to be at a substantial disadvantage [21].. This may help to explain why countries like India have sought to adopt clear statutory bars on certain types of secondary patent claims, even if those bars are not always consistently implemented during patent examination [28].

Our data also reveal the stakes of the decision that developing countries must make about the permissible scope of patents. Although the World Trade Organization's Trade-Related Aspects of Intellectual Property Agreement does require member countries to adopt patent protection for medicines, its requirements are general, and do not clearly require countries to permit secondary patents [21]. We can quantify the stakes of such decisions: If the future looks like the past (and the patent landscape in other countries like that in the U.S.) a conservative estimate is that eliminating secondary patents could free up 36% of new medicines for generic production, since only 64% of drugs in our sample had patents with chemical compound claims. Additionally, for those drugs that still come under patent because a chemical compound claim exists, exclusions on secondary patents could limit the duration of patent protection by 4–5 years. The converse is that this study reveals the very substantial implications of new trade agreements. Negotiations are now underway for a new “Trans-Pacific Partnership” treaty, and the U.S. has apparently proposed barring exactly the kind of limits on secondary patents adopted by India, and under consideration by other countries.

Finally, our data also have relevance to the evolution of patent law in developed countries. Recent court decisions in the U.S. have seemed to signal a more restrictive approach to at least certain secondary patents in the U.S. [29]. While we do not here address whether such a change would on balance do more to harm patient health (by undermining innovation) than to help (by improving access), we do clarify the very substantial stakes of this debate.

While the data provided here can be interpreted in different ways, it should, we think, advance the policy debate in several ways. Most importantly, it should make clear that secondary patents are of substantial importance in the industry, and that analyses that focus only on chemical compound patents will tend to substantially underreport both the breadth and range (term) of patent coverage in the pharmaceutical sector.

## Supporting Information

Table S1 **Description of patent claim categories.**
(DOCX)

Appendix S1 **Description of patent claim categories.**
(DOCX)
