# Supplementary material for: Polymorphs and Prodrugs and Salts (Oh My!): An Empirical Analysis of “Secondary” Pharmaceutical Patents
Source: PLoS One. 2012 Dec 5;7(12):e49470. doi: 10.1371/journal.pone.0049470 (PMC3515607; doi:10.1371/journal.pone.0049470)
Supplement: Table S1 — Description of patent claim categories. (DOCX) [file pone.0049470.s001.docx]

**Supplementary Table 1: Description of patent claim categories**

| **PRIMARY** | | |
| --- | --- | --- |
| **Claim category** | **Description** | **Notes** |
| Chemical Compound | Includes active ingredients that had not previously been disclosed in the art.  Excludes claims covering new forms of a previously disclosed compound (coded separately as belonging to one of the "secondary" categories below). | - In the patent literature these generally referred to as "compound claims." - Patents with these claims correspond roughly with what the European Commission has termed “primary patents,” in that they are generally the “first ever patent covering a particular pharmaceutically active ingredient.” [6, Annex B, Page 299] However, this correspondence is not perfect. For example, in our sample, an active ingredient is sometimes associated with more than one chemical compound claim. This may be because an initial patent can claim a broad class of chemical compounds, and a later patent can claim a selected compound or subset of compounds within that class. |
| **SECONDARY** | | |
| **Claim category** | **Description** | **Notes** |
| Formulation | Includes specific pharmaceutical formulations or compositions. Such claims may cover, for example, a tablet, parenteral or suspension form, a sustained release form, or a particular dosage (once-a-day, with food, etc.).  Excludes claims directed specifically at combinations of two active ingredients. | - Pharmaceutical products are almost never administered solely as an active ingredient, because the active ingredient may be a fraction of a milligram (and so nearly invisible), and/or have physical properties (heat or light sensitivity, liquid form) that make independent administration difficult [8]. - A typical formulation claim covers “a pharmaceutical composition, comprised of [active pharmaceutical ingredient] combined with a pharmaceutically acceptable carrier” [6, page 601] |
| Method of treatment / use | Includes claims for “method of treating" a disease Y with a specific compound X or "the use of compound X for treatment of disease Y." | - These claims are sometimes part of patents with chemical compound claims, but also often form the primary subject matter of patents, particularly where the product itself cannot be claimed independently due to lack of novelty. - Patents with these claims can be used to obtain protection for new uses of known substances. |
| *PIPES (Polymorph, Isomer, Prodrug/Ester, Salt) Secondary Claims* | | |
| Polymorph | Includes polymorph, solvate, hydrate, and crystalline forms, when one or more specific forms are claimed.  Excludes generic claims covering all or a very large set of possible forms. | - Pharmaceutical substances often exist in two or more solid-state forms “Crystalline” forms have different arrangements and/or conformations of the molecules in the crystal lattice. Amorphous forms consist of disordered arrangements of molecules that do not possess a distinguishable crystal lattice. Solvates and hydrates are crystal forms that have a solvent (in case of hydrates, water) incorporated into their crystal structure [26]. |
| Isomer | Includes isomers and enantiomers, where these configurations were identified and isolated from a previously known compound. | - A molecular compound with the same empirical formula can often exist in various structural configurations, called isomers. - Enantiomers are specific types of isomers representing pairs of molecules that are mirror images of one another and not superimposable [28]. Despite sharing an identical molecular formula, the different configurations of enantiomers can cause them to exhibit different physical or chemical properties - Sometimes, one specific enantiomer of a drug molecule exhibits more favorable characteristics. Thus, it is not uncommon for an earlier patent application to cover the racemic mixture and a later patent to claim a specific enantiomer. A well-known example is esomeprazole (marketed as Nexium by AstraZeneca), which is a single enantiomer of a previously approved drug, omeprazole (marketed as Losec/Prilosec). |
| Prodrug/Ester | Includes esters, prodrugs, and ester prodrugs. Since these claims are often indistinguishable from chemical compound claims, we identified these not only by reading patent claims, but also by reviewing lists of prodrugs available in the secondary literature. | - Esters and prodrugs represent minor modifications of existing drugs. - Esters include chemical compounds most commonly derived from carboxylic acids by reaction with a hydroxyl compound (such as an alcohol or phenol). - While esters can be pharmaceutically active, prodrugs are compounds that are inactive, but are metabolized in the body to form an active drug [10]. - Prodrugs may occur naturally, or may be “formed by intentionally linking a drug to an inert chemical by a covalent bond, which may be broken…to yield the drug itself in vivo” [30, page 25]. This is most commonly done when the active agent is unable to be absorbed into the blood stream in sufficient quantities [6] - One type of prodrug compound is ester prodrug, which is hydrolyzed to an active carboxylic acid-containing drug [31]. (An example is the antiviral acyclovir, which was later developed into the prodrug valacyclovir, which is independently administered and more bioavailable than acyclovir [10].) |
| Salt | Includes claims directed to a specific salt form, or a small genus of specific salt forms.  Excludes generic claims for "all pharmaceutically acceptable salts." | - The benefits of converting drug substances into a particular salt form is known throughout the pharmaceutical industry: salts tend to yield better bioavailability, stability and manufacturability characteristics, without modifying the active compound’s chemical structure [32, 33]. |

Note: Other patent claims (process claims, product-by-process claims, medical device claims, particle sizes, combinations, and pure forms) excluded from analyses of secondary patenting since small in number or not of substantial importance in analyses of secondary patenting.
